# Detecting fake news on Facebook: The role of emotional intelligence

**DOI:** 10.1371/journal.pone.0246757

**Published:** 2021-03-11

**Authors:** Stephanie Preston, Anthony Anderson, David J. Robertson, Mark P. Shephard, Narisong Huhe

**Affiliations:** 1 School of Psychological Sciences and Health, University of Strathclyde, Glasgow, United Kingdom; 2 School of Government and Public Policy, University of Glasgow, Glasgow, United Kingdom; University of Malaga, SPAIN

## Abstract

The proliferation of fake news on social media is now a matter of considerable public and governmental concern. In 2016, the UK EU referendum and the US Presidential election were both marked by social media misinformation campaigns, which have subsequently reduced trust in democratic processes. More recently, during the COVID-19 pandemic, the acceptance of fake news has been shown to pose a threat to public health. Research on how to combat the false acceptance of fake news is still in its infancy. However, recent studies have started to focus on the psychological factors which might make some individuals less likely to fall for fake news. Here, we adopt that approach to assess whether individuals who show high levels of ‘emotional intelligence’ (EQ) are less likely to fall for fake news items. That is, are individuals who are better able to disregard the emotionally charged content of such items, better equipped to assess the veracity of the information. Using a sample of UK participants, an established measure of EQ and a novel fake news detection task, we report a significant positive relationship between individual differences in emotional intelligence and fake news detection ability. We also report a similar effect for higher levels of educational attainment, and we report some exploratory qualitative fake news judgement data. Our findings are discussed in terms of their applicability to practical short term (i.e. current Facebook user data) and medium term (i.e. emotional intelligence training) interventions which could enhance fake news detection.

## Introduction

The dissemination of misinformation has always been a feature of society [1–4]. However, the ubiquity and potential damage that such misinformation, or ‘fake news’, can have, has been elevated significantly by the emergence of social media platforms such as Facebook, which engages a global audience [5]. The focus on the role of fake news in the 2016 UK EU Referendum [6,7], the 2016 US Presidential Election [8,9], and the 2020 COVID-19 pandemic [10,11] have become matters of considerable public and governmental concern.

Fake news can take several forms, ranging from instances in which visual or textual information is inserted into an article in order to subtly bias an argument one way or the other, to wholly fabricated content, often including extraordinary claims, which is created and shared systematically with the sole intention to deceive [12,13]. Fake content captures attention and spreads rapidly in comparison to real news [14], and such items often employ emotionally charged language [15]. Research suggests that such emotional content could be one of the key factors which prevents social media users from engaging in a critical assessment of the core message [16,17]. However, one psychological concept, known as ‘emotional intelligence’ (EQ) [18,19], could attenuate this effect, and in this paper we assess whether users who exhibit higher levels of emotional intelligence are better able to detect and discard fake news content.

One of the engaging aspects of social media is the rapidity with which news and information is updated, new posts from information content providers compete with each other to create highly salient ‘bite sized’ headlines which can be rapidly digested, before moving on to the next item [20,21]. This format is well suited to users who wish to be presented with fresh content which reflects their existing attitudes, fears, and implicit prejudices [22,23], and it capitalises on their existing emotional connections to particular topics (e.g. political affiliation). In contrast, this type of news platform is ill suited to the type of critical thinking and analytical reasoning that is required to judge the truthfulness of content [24,25]. It is no surprise therefore that emotionally salient content is most likely to capture a user’s interest and, given the short focus of their attention, bias or confirm their opinion in the way that fake new providers intend [22,23,26].

Indeed, research from the wider cognitive psychology literature supports the view that emotional or affective information is processed rapidly, and in some cases in the absence of focused attention [27,28]. In addition, studies assessing higher cognitive processes have shown that emotion does influence decision making, judgement formation, and risk perception [29–32]. Although these studies did not directly assess the effects of emotional content on the detection of fake news, they did support the development of models of emotional intelligence (EQ), and they suggest that dealing with emotional experiences is a process which relies on several different characteristics [18,19,33–37].

Such characteristics include being able to accurately perceive and reflect on emotional content, to make correct links between emotion and context, and the ability to regulate one’s own emotional reactivity. These interdependent skills are ranked in terms of higher-order processes (i.e. strategic-EQ, understanding and processing of emotions) and lower-order processes (i.e. experiential-EQ, perception and process) [19,38]. Individuals who score highly in EQ should be better at understanding and regulating their own emotions, and importantly, the emotional content of news items that appear on their social media feed. Therefore, in the present study, we investigate whether individuals who exhibit high levels of emotional intelligence (i.e. high EQ) are more likely to see through the emotionally charged content found in fake news items. If that is the case, such individuals should be able to devote more of their cognitive resources to the type of analytical reasoning and critical thinking which would allow them to identify fake news content, resulting in higher fake news detection scores [see 20,21].

**Fig 1:**
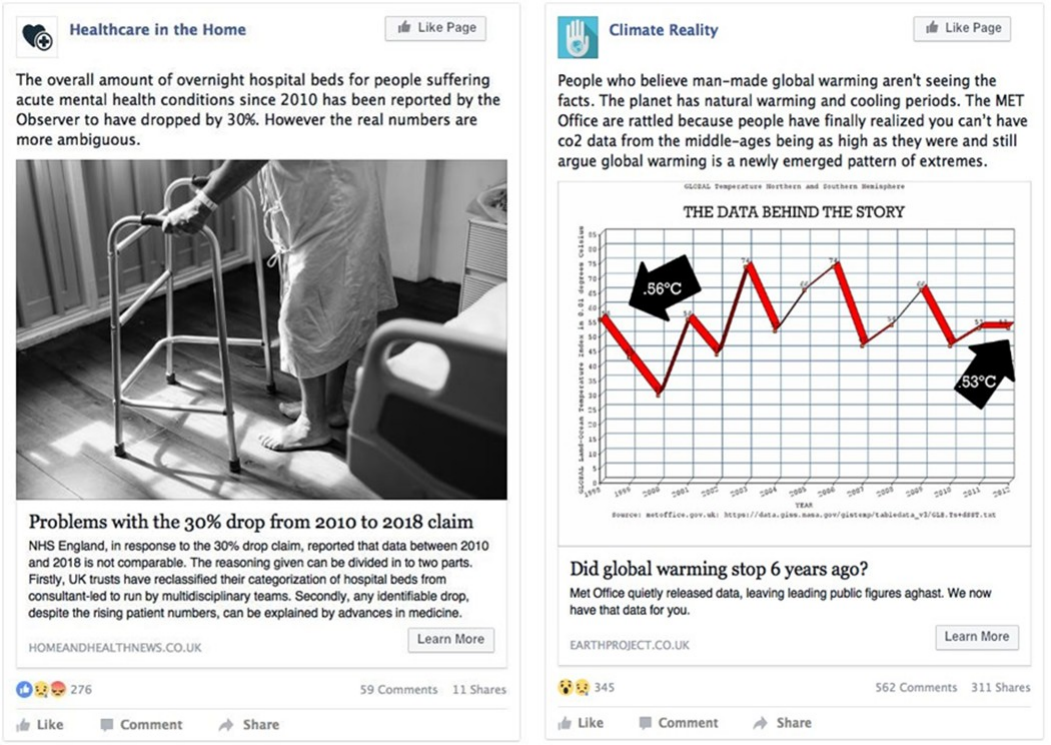
Shows an example of a real news item on the left and a fake news item on the right. Participants assessed each item in relation to their perception of its objectivity, professionalism, argument strength and trustworthiness/credibility, and then they were given an opportunity to explain their responses via a text input box.

To that end, here we present participants, recruited from the UK, with a novel version of the fake news detection task reported in Pennycook and Rand (2018a) [21], in which real and fake news items are displayed in a typical Facebook format with high ecological validity, an example trial is presented in Fig 1. Our study makes a novel contribution to the growing fake news literature in four important ways. First, using an established measure of EQ [33] we assess whether individual differences in emotional intelligence are associated with fake news detection aptitude. Second, participant’s responses to our news items combine their judgements of the item’s objectivity, professionalism, argument strength and overall trustworthiness/credibility, rather than the 2AFC (i.e. is this real or fake news) used in previous research. Third, research has shown that emotional intelligence is positively correlated with academic achievement [39–41], and so here we seek to replicate that effect and assess whether it extends to fake news detection ability. Fourth, we capture and report qualitative response data on how and why participants have arrived at their judgements, and we perform an exploratory analysis which highlights the potential reasoning behind the acceptance or rejection of our fake news item set.

## Materials and methods

### Ethics statement

This study was approved by the Ethics Committee of the University of Strathclyde School of Psychological Sciences and Health. All participants provided written informed consent.

### Participants

An a-priori G*Power analysis with an assumed medium effect size of.30, power set at.80 and an alpha of.05, suggested that a sample size of 67 participants was required to detect an effect in this study. Therefore, to ensure adequate statistical power, we recruited 104 participants for this study. 17 participants were removed from the final dataset as they did not complete all aspects of the study, and so the final sample consisted of 87 participants (55 Female). The majority of participants in the sample were aged between 17 and 35 (*N* = 66) with the remaining participants aged between 36 and 56 (*N* = 21). Note, that in Scotland, where the data was collected, the age of majority is 16, and so the individuals included in our sample who were aged 17 were able to provide informed consent as adults. Participants were recruited via the Strathclyde Psychology Participant Pool and via a Qualtrics link posted on social media (@UOSPsychology).

We use the full sample of 87 participants to assess the effects of EQ on fake news detection, and in addition we split the participants into two groups on the basis of their level of educational attainment to assess the same outcome. Group 1 included those who had completed, or were in the process of completing, a university degree, there were 57 participants in this group (40 Female) and the majority aged between 17 and 35 years (*N* = 50) with a small proportion aged between 36 and 56 years (*N* = 7). Group 2 included those who had a school/UK college level education, there were 30 participants in this group (15 Female) with half aged between 17 and 35 years (*N* = 16) and half aged between 36 and 56 years (*N* = 14). While the two groups are not equally matched in terms of sample size, we include these groups and this analysis to assess any indicative effects of educational attainment on fake news detection. All participants were naïve to the purpose of the study, had normal or corrected-to-normal vision, and participants who were recruited from the psychology participant pool received a course credit on completion of the study to reimburse them for their time.

### Measures

#### Emotional intelligence

The Emotional Intelligence Questionnaire [33], a public domain resource obtained from https://ipip.ori.org/, has 7 components, but for the purposes of the present study we selected the three which best complemented the design of our critical thinking task. That is, we selected those subscales which had greater focus on self-awareness, self-regulation and empathy, rather than those measuring dimensions of social skill, mood, and relationship-handling. Our EQ measure therefore comprised of items relating to; Attending to Emotions (ATE; 10 items), Emotion-Based Decision-Making (EBDM; 9 items) and Empathic Concern (EC; 10items). The measure consisted of 29 items in total, with each item being presented with a 5-point Likert response scale which ranged from 1 (strongly agree) to 5 (strongly disagree). See [33] for the items that were reverse scored. The minimum score on this measure was 29 while the maximum possible score was 145—*lower scores indicate higher levels of EQ*. The Cronbach’s alpha for each component demonstrates high internal consistency; Attention to Emotion = .83, Emotion Based Decision Making = .67 and Empathic Concern = .80.

#### News items and fake news detection task

*News item set*. We created a bespoke Fake News Detection Task which was developed to tap into the critical analysis skills which might allow an observer to ascertain whether an item is likely to be real or fake. The task was based on that reported in Pennycook and Rand’s (2018a) [21] study on fake news susceptibility, where participants were presented with news items in the style and format common to Facebook. However, while Pennycook & Rand’s (2018a) [21] task focused on politically charged topics (e.g. Democratic/Republican politics), here we chose to avoid any overtly political statements in favour of more emotionally salient topics.

There were six news items in total, three presented real news content and three presented fake news content. For the real news items, the news topics related to: the reduction of numbers of beds in mental hospitals for overnight patient stay (real news), the use of children by criminal gangs to distribute drugs (real news), the reporting by government of underestimated immigration figures (real news). For the fake news items, the news topics related to: a claim that large numbers of NHS patients are discharged from hospital at night (fake news), a claim that students from less wealthy backgrounds are unable to participate in university education (fake news), and a claim that global warming effectively ceased six years ago (fake news). Information for all items was sourced through the independent and impartial fact-checking network, www.fullfact.org, a free to use public domain fact checking resource.

*Facebook post item format*. Four main components of the mock Facebook posts were considered during item development to increase participants’ opportunities to evaluate levels of objectivity, professionalism, argument strength and trustworthiness in the items. These four components were *News Sharing Source*, *Original News Item Source*, *Content Level*, *and Author Argument*.

For component 1, *News Sharing Source*, our items depicted typical Facebook news posts in which an article was being shared by an organisation related to its content, as seen in Fig 1, the Facebook page ‘Healthcare in the Home’ is sharing an item from ‘Homeandhealthnews.co.uk’. All of the sharing source names were chosen to influence impressions of professionalism and objectivity. More subjective-sounding content names were used for fake news items (i.e. ‘Have your say’, ‘Student Source Daily’, and ‘Climate Reality’) and more objective content names for real news items (i.e. ‘Healthcare in the Home’, ‘Community Dialogue’, and ‘Understanding the Numbers’). For component 2, *Original News Item Source*, this relates to the actual source of the article not the page that is sharing it. Fake news items had source web-addresses that suggested more subjective content (i.e. Health Thoughts, Student-Talk-And-Share.org, and EarthProject.co.uk), whereas, fact-driven items displayed web address names suggestive of more objective content (i.e. HomeAndHealthNews.co.uk, SafetyAndAawreness.org and NumericalNews.co.uk).

For component 3, *Content Level*, the fake news items only included brief information, without sources, written more subjectively with the aim being to suggest low trustworthiness. For component 4, *Author Argument*, the author arguments for fake news items were written to include; emotive language to suggest subjectivity (e.g. ‘I’m more angry at the lack of answers than anything, saying the numbers doesn’t tell us enough’), plus a lack of any references to credible sources to suggest low levels of professionalism, argument strength, and trustworthiness. For real news items the opposite strategies were employed, these included non-emotive language and credible sources (e.g. ‘The National Crime Agency (NCA) has found that children, under the age of 12, have been exploited by adults to distribute drugs’).

In order to ensure a high level of ecological validity in each post and to suggest engagement with the articles from other Facebook users, as seen in Fig 1 each post included the typical Facebook displays relating to likes, comments and shares. Numbers of likes, comments and shares varied randomly across items but did not vary systematically between real and fake item groups.

*Fake news detection task*. For each of the six news items, participants were required to critically evaluate each article and respond to four questions, responses were made via a 5 point Likert scale which ranged from 1 (strongly agree) to 5 (strongly disagree). Each question was prefaced with the text ‘to what extent do you agree with the following statement’: Q1 (Objectivity) ‘the author and attached article are objective’, Q2 (Professionalism) ‘the article looks to be produced by a professional’, Q3 (Argument Strength) ‘the article presents a strong argument’, and finally the explicit fake news detection question, Q4 (Trustworthiness) ‘this source of information is credible and trustworthy’. Responses to the fake news items were reverse scored. As a result, scores on the Fake News Detection Task could range from 24–120, with the lowest score, 24, representing the strongest fake news detection performance and the highest score, 120, representing the weakest performance. Participants’ responses revealed that the fake news detection measure had a high degree of internal reliability with a Cronbach’s alpha = .92. For each item, upon completing the four questions noted above, participants were given the opportunity to explain their responses via a text entry box, this provided qualitative response data.

### Procedure

The task was presented to participants using Qualtrics, the online testing platform, and this software was also used to collect the data. Participants initially read an information sheet describing the study and provided informed consent by clicking the appropriate onscreen response. Participants were then presented with the tasks in a fixed order: EQ Task, Fake News Detection Task, and they then reported their level of educational attainment. For the Fake News Detection Task, participants were instructed to critically analyse each news item and to respond to each of the four questions. Upon completion of these measures, all participants received the experimental debrief which, importantly, made them aware of which items were real and which were fake.

## Results

### Fake news detection & EQ scores

Scores for the Fake News Detection Task, overall EQ, and each of the EQ subtestsare reported in Table 1. The midpoint score for the detection task, which would be indicative of ‘don’t know’ responses was 72, and as seen in Table 1, overall detection scores were 61. This confirms that our item manipulation was successful, and while the task, like real world fake news detection, was challenging, participants were more likely to correctly detect the fake news items than not. Moreover, as seen in Table 1, there was a large degree of variability both in fake news detection and EQ scores, which confirms that the following individual difference analysis in which we assess whether EQ is associated with fake news detection is statistically valid.

**Table 1:** Summary scores.

	Mean	SD	Range
Fake News Detection	61	18	25–120
Overall Emotional Intelligence (EQ)	71	22	42–137
Attention To Emotion (EQ-ATE)	22	10	10–50
Emotion Based Decision Making (EQ-EBDM)	27	7	12–45
Empathic Concern (EQ-EC)	21	8	11–46

Summary scores for the fake news detection task and the emotional intelligence measure (overall, and by subtest).

### Emotional intelligence and fake news detection

**Fig 2:**
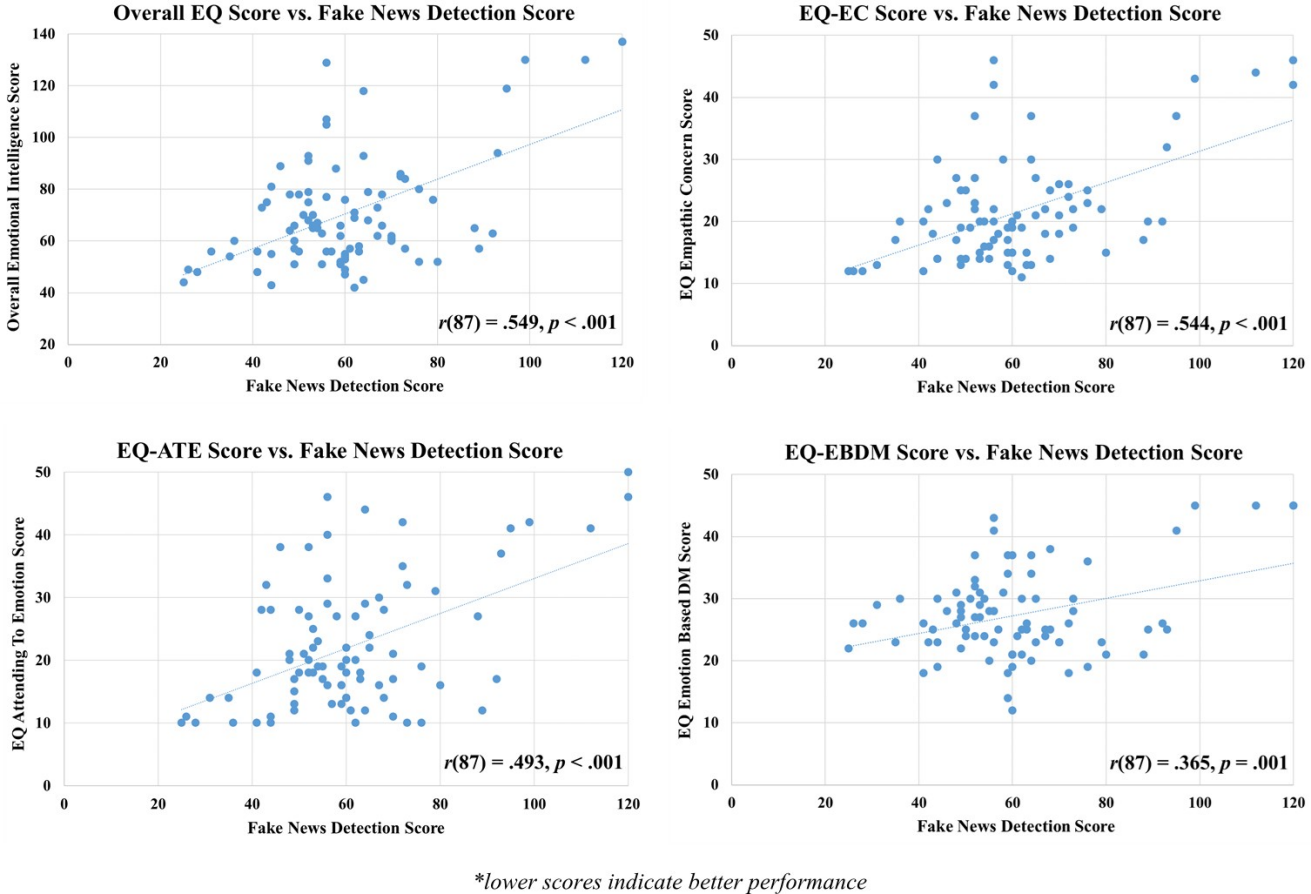
Scatterplots showing correlations between fake news detection scores and scores on the emotional intelligence questionnaire (Overall EQ), and each of its three subtests (EC—Empathic Concern; ATE—Attending to Emotions; EBDM—Emotion Based Decision Making).

Pearson’s correlation analysis was used to test for associations between scores on the Fake News Detection Task and overall and subtest EQ scores. As seen in Fig 2, statistically significantly positive correlations, of moderate effect sizes, were found between fake news detection scores and overall EQ scores, as well as for each of the EQ subtests (these effects remained significant after applying the Bonferroni correction, new alpha level = .01). This finding shows that the more emotionally intelligent a participant was, the less likely they were to fall for fake news.

### Educational attainment and fake news detection

**Fig 3:**
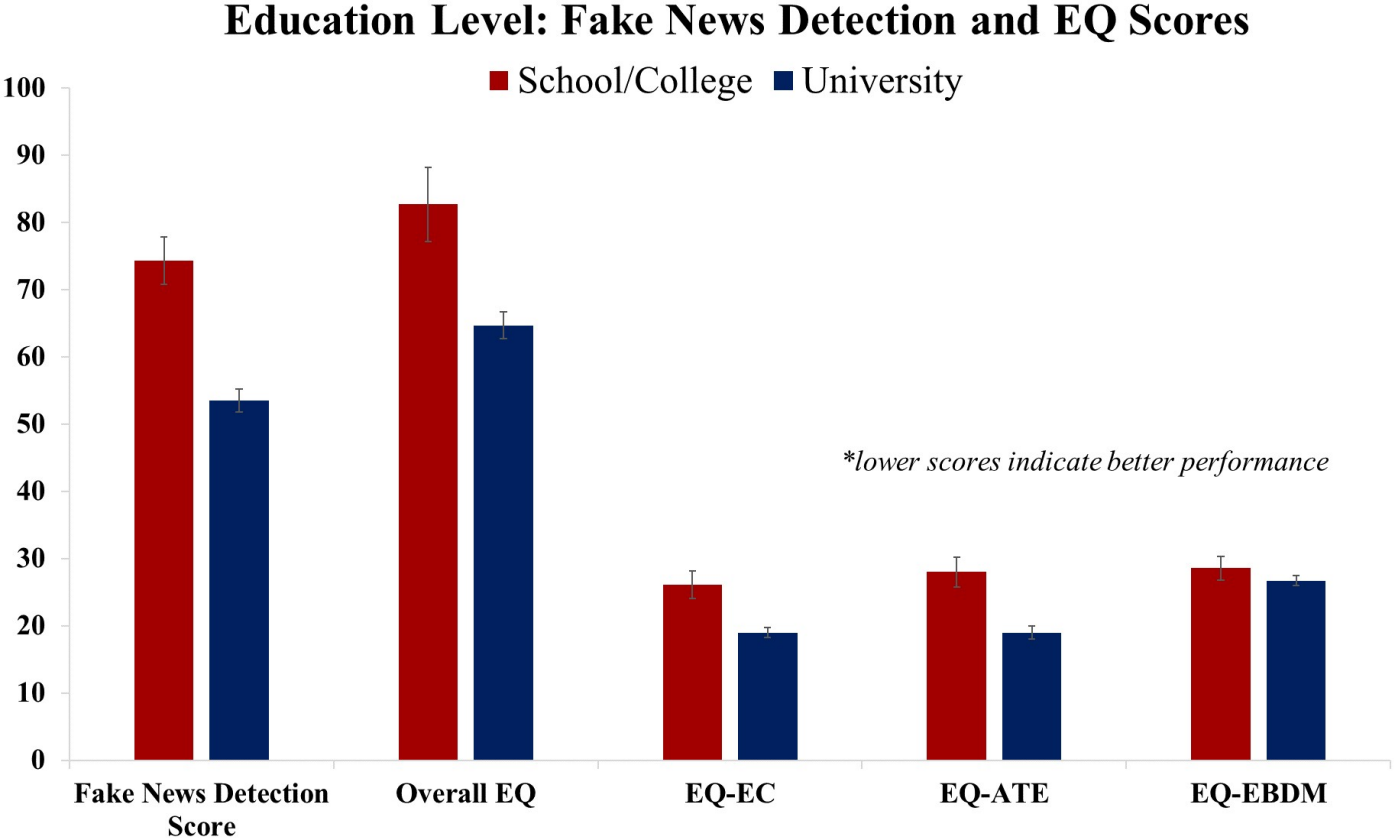
Fake news detection scores, overall and subtest emotional intelligence scores, presented as a function of educational group (school/college educated, university educated; error bars denote standard error of the mean).

Independent samples t-tests revealed that university educated participants scored significantly better on the fake news detection tasks, *t*(85) = 6.03, *p* < .001, as well as on the overall measure of emotional intelligence, *t*(85) = 3.87, *p* < .001, and two out of the three EQ subtests (*t*(85) = 4.30, *p* < .001 for EQ-ATE; *t*(85) = 4.09, *p* < .001 for EQ-EC; *t*(85) = 1.17, *p* = .246 for EQ-EBDM), than their school/UK college educated counterparts. Summary data presented as a function of educational group is shown in Fig 3, lower scores on all measures indicate better fake news detection performance and greater levels of emotional intelligence.

### Qualitative response analysis for fake news items

Here we provide a sample of the text responses to the final question which appeared on each trial ‘*Please briefly describe the reason(s) for your answers*’. As the intention is to assess, from qualitative responses, why some participants might have fallen for the fake news items and why others might have correctly detected them, we collapsed the data across the three fake news items and included responses only from participants who had provided a clear opinion (i.e. strongly agree orstrongly disagree) to their opinion of the overall trustworthiness and credibility of the item (Q4).

As seen in Table 2, four themes emerged which best captured the text responses of those who fell for the fake news items, and six themes emerged for those who correctly rejected the fake content. For those participants who indicated that they thought the fake news items were trustworthy and credible, the themes that emerged related to the item apparently corroborating personal experience, the inclusion of data and graphs, that the item was bringing to light an important but previously ‘suppressed’ problem, and that they were in agreement with the point being made as it fit their existing beliefs. For those participants who indicated that these items were not credible and trustworthy (i.e. they were likely to be fake news), the themes that emerged related to the obvious use of overly emotive language, the lack of supporting data or facts, concern over the unofficial nature of the news source, the unprofessional ‘opinionated’ tone used by the post author, graphs and visuals that looked unprofessional, and the fact that the content contradicted their own understanding of current facts and science (e.g. in relation to climate change).

**Table 2:** Qualitative data themes.

**Those who incorrectly accepted the fake news content**	**Theme**
I have personal experience of this	**Personal Experience**
My kids are in this position so I completely get this	
Good argument, it uses a graph and data from a source	**Visuals/Data/Graphs**
The graph shows it all	
This calls out poor practice and makes sense	**A ‘Hidden’ Problem**
Horrible problem, glad it was pointed out	
The commenter on the post has the same thoughts as me	**Fits Existing Beliefs**
Agree, makes good points	
**Those who correctly rejected the fake news content**	**Theme**
Emotive language, subtitle is designed to create a reaction	**Emotive Language**
There is emotive/condescending language in the blurb	
Fearmongering article with no data	**No Supporting Data**
No data/facts to back up the information	
The source is not an official scientific, or governmental source	**Source Concerns**
It doesn’t look official/look like it is from a trustworthy source	
Comes across as more of a rant	**Unprofessional Tone**
From an opinion page, talking in the first person	
The graph looks bad	**Poor Graphs/Visuals**
The data is not presented well	
There is proof that we are causing global warming	**Appeals To Evidence**
Climate change is real	

Shows the general themes that emerged from the text responses to the question which appeared at the end of the list for each news item ‘Please briefly describe the reason(s) for your answers’. Here we present themes for the fake news items only.

## Discussion

This is, to our knowledge, the first paper to assess whether fake news detection ability is associated with individual differences in emotional intelligence, and UK levels of educational attainment (see [42] for recently published related work in the U.S.). Using a novel and comprehensive measure of fake news detection ability, which combined judgements of objectivity, professionalism, argument strength and overall trustworthiness/credibility, participants were presented with three fake and three real news items. For overall fake news detection scores, our findings show that while the task was challenging, participants were, on average, more likely to detect the fake news items than not. The task produced no floor or ceiling effects, and there was a good level of variation in detection performance and EQ scores.

Importantly, our findings show that individual differences in fake news detection scores were associated with individual differences in overall emotional intelligence. That is, participants who were more emotionally perceptive were less likely to fall for fake news. This finding supports the idea that high-EQ individuals are more likely to be able to see beyond the emotionally charged content which is a hallmark of fake news, leading to a more effective critical evaluation of the likely veracity of the content. Here we used fake news detection scores as a proxy for critical thinking aptitude. We can conclude that high-EQ individuals were better at evaluating fake news content, however, as we did not include a direct measure of critical reasoning, it is not clear whether high-EQ individuals are also better critical thinkers *per se*. It might be the case that low-EQ individuals would perform equally well on non-emotion based tests of fake news detection. In order to assess this directly, we suggest a follow up study which adds an established measure of critical analysis skills such as the Cognitive Reflection Test (CRT) [43,44] to the current battery, this is something that we now intend to pursue.

However, research has shown that critical thinking skills are positively correlated with academic achievement [39], and here we show that fake news detection performance was better in participants who had, or who were in the process of obtaining, a university degree in comparison to their school/UK college level counterparts. Moreover, we also replicate previous work which shows that academic achievement is also positively related to levels of emotional intelligence [40,41]. Taken together, these findings suggest that, in the absence of a direct measure of critical thinking skills such as the CRT, it is likely that high-EQ individuals may indeed also be better critical thinkers. This work therefore also has wider implications regarding theoretical models of Critical Thinking [e.g. 44], which often overlook affective content to focus on the cognitive aspects of argument analysis and evidence use. While our group distinction did replicate and extend previous work, this effect of education on fake news detection must be treated cautiously until it is replicated with larger samples in which the groups are equally matched on a wide range of non-educational demographics.

While studies of fake news detection have predominantly focused on assessing task accuracy and associations between detection performance and other psychological measures, here we extended this methodology to incorporate qualitative data. Our findings showed that a number of clear themes emerged from this analysis, with those that supported the incorrect acceptance of fake news items speaking to issues of personal experience, existence of graphs/data, uncovering a ‘hidden problem’, and the item fitting with existing beliefs. Taken together, these themes match the idea that confirmation bias [26] is a powerful component which may underpin the false acceptance of fake news. In contrast, participants who judged the fake news items as low on creditability and trustworthiness tended to focus on the absence of official sources, data, unprofessional tone and format, and their own understanding of existing science/facts as hallmarks with which to question the content of such items. Future studies should seek to incorporate a qualitative approach into their design which would yield content for a full and comprehensive thematic analysis. In doing so, should the same themes arise, these could be developed into a training technique to enhance a user’s ability to detect fake content. In addition, our sample consisted primarily of young adults, future research should also focus assessing fake news detection across a wider age range [45,46].

The findings from this study also suggest further training routes and initiatives that could enhance fake news detection. First, research has shown that it is possible to train individuals to enhance their levels of emotional intelligence, if such training could be incorporated into fake news detection initiatives presented to students in secondary education, this could enhance their ability to discard misinformation as they approach the age at which they will become social media users [see 47,48]. Second, and perhaps a more immediate intervention, would be to capitalise on the work of Kosinski and colleagues [49,50] who showed that they could deduce a user’s psychological profile simply by looking at their Facebook data. If a similar approach could be used to rate a person’s level of emotional intelligence from such data then Facebook could alert low EQ scores that they should be more vigilant about misinformation and fake news that might appear on their platform.

## Conclusions

To conclude, here we present a small-scale exploratory study which assessed whether there were associations between fake news detection, emotional intelligence, and educational attainment. Using a novel measure of fake news detection, we show that individuals who are high in emotional intelligence and who are in receipt of a university education are less likely to fall for fake news than low EQ/School-College educated individuals. Our qualitative findings also provide more depth and insight into why some people fall for fake news when, for example, the item content fits their pre-existing beliefs. Finally, we outline ways in which our findings could be used to enhance fake news detection in the short (i.e. using existing Facebook user data) and medium terms (i.e. emotional intelligence training).

## Supporting information

S1 File Summary data. (XLSX)
