# Correction: Detecting fake news on Facebook: The role of emotional intelligence

**DOI:** 10.1371/journal.pone.0258719

**Published:** 2021-10-13

**Authors:** Stephanie Preston, Anthony Anderson, David J. Robertson, Mark P. Shephard, Narisong Huhe

There is an error in affiliation 2 for authors Mark P. Shephard and Narisong Huhe. The correct affiliation 2 is: School of Government and Public Policy, University of Strathclyde, Glasgow, United Kingdom.
